# Blood Leukocyte *AHRR* Methylation and Risk of Non–smoking-associated Cancer: A Case-cohort Study of Non–Hodgkin Lymphoma

**DOI:** 10.1158/2767-9764.CRC-23-0151

**Published:** 2023-09-07

**Authors:** Christina Dahl, Ulla A. Hvidtfeldt, Anne Tjønneland, Per Guldberg, Ole Raaschou-Nielsen

**Affiliations:** 1 Molecular Diagnostics, Danish Cancer Institute, Copenhagen, Denmark.; 2 Work, Environment and Cancer, Danish Cancer Institute, Copenhagen, Denmark.; 3 Diet, Cancer and Health, Danish Cancer Institute, Copenhagen, Denmark.; 4 Department of Public Health, University of Copenhagen, Copenhagen, Denmark.; 5 Department of Cancer and Inflammation Research, Institute for Molecular Medicine, University of Southern Denmark, Odense, Denmark.; 6 Department of Environmental Science, Aarhus University, Roskilde, Denmark.

## Abstract

Aryl-hydrocarbon receptor repressor (*AHRR*) hypomethylation in peripheral blood is tightly linked with tobacco smoking and lung cancer. Here, we investigated *AHRR* methylation in non–Hodgkin lymphoma (NHL), a non–smoking-associated cancer. In a case-cohort study within the population-based Danish Diet, Cancer and Health cohort, we measured *AHRR* (cg23576855) methylation in prediagnostic blood from 161 participants who developed NHL within 13.4 years of follow-up (median: 8.5 years), with a comparison group of 164 randomly chosen participants. We measured DNA-methylation levels using bisulfite pyrosequencing and estimated incidence rate ratios (IRR) using Cox proportional hazards models with adjustment for baseline age, sex, educational level, smoking status, body mass index, alcohol intake, physical activity, and diet score. Global DNA-methylation levels were assessed by long interspersed nucleotide element 1 (LINE-1) analysis. Overall, the IRR for *AHRR* hypomethylation (lowest vs. other quartiles) was 2.52 [95% confidence interval (CI), 1.24–5.15]. When stratified according to time between blood draw and diagnosis, low *AHRR* methylation levels were associated with a future diagnosis of NHL [IRR: 4.50 (95% CI, 1.62–12.50) at 0–<5 years, 7.04 (95% CI, 2.36–21.02) at 5–<10 years, and 0.56 (95% CI, 0.21–1.45) at ≥10 years]. There was no association between global DNA-methylation levels and risk of NHL. Our results show that *AHRR* hypomethylation in blood leukocytes is associated with a higher risk of NHL in a time-dependent manner, suggesting that it occurs as a response to tumor development.

**Significance:**

Our population-based study demonstrated that lower *AHRR* methylation levels in peripheral blood leukocytes were associated with an increased risk of NHL. This association was independent of tobacco smoking, sex, and lifestyle characteristics, but was highly dependent on time to diagnosis. These findings highlight the potential of *AHRR* methylation as a biomarker for NHL risk, effective up to 10 years after blood draw.

## Introduction

Alterations in DNA methylation patterns represent a hallmark of cancer (1). DNA methylation is a covalent modification that occurs at cytosine nucleotides positioned in front of a guanine (CpG dinucleotides). Hypermethylation of DNA may lead to silencing of gene transcription when occurring within promoter regions and when occurring in gene bodies and noncoding regions, may stabilize the DNA structure and prevent movement of transposable elements such as long interspersed nuclear elements (LINE). Studies on DNA methylation in cancer have predominantly examined tumor biopsies, revealing changes that are limited to cancerous cells (2). However, several studies have identified altered DNA methylation signatures in peripheral blood leukocytes from patients with solid cancers (3–5) and suggested that these signatures may predict a future diagnosis of cancer in seemingly healthy subjects (6).

Whole-methylome sequencing has identified many individual CpG sites that are differentially methylated in peripheral blood leukocytes of patients with cancer as compared with healthy individuals. The most extensively characterized of these CpG sites are in intron 3 of the gene encoding the aryl-hydrocarbon receptor repressor (*AHRR*) and include cg05575921 and cg23576855. Hypomethylation of these sites in blood leukocytes is strongly correlated with tobacco smoke exposure (7–13), and methylation levels recover in individuals who quit smoking (14). *AHRR* hypomethylation is also associated with lung cancer and, interestingly, predicts lung cancer diagnosis independent of smoking status (12, 15–17). To our knowledge, no studies to date have examined the association of *AHRR* methylation with other cancers.

Although the association between *AHRR* hypomethylation and lung cancer has been replicated in several independent cohorts, it remains unknown whether altered *AHRR* methylation is causally involved in lung cancer development or represents an early response to tumor (18). A study employing Mendelian randomization suggested that low blood levels of *AHRR* methylation are not causally linked to lung cancer (18). On the other hand, a large study employing mediation analysis in four prospective cohorts suggested that a considerable part (>30%) of the effect of smoking on lung cancer was mediated by changes in *AHRR* methylation (12). A proposed mechanism is that *AHRR* hypomethylation leads to increased expression of the *AHRR* in immune cells such as monocytes, macrophages, and lymphoblasts, hampering their capability of metabolizing toxic or carcinogenic substances contained in tobacco smoke (12).

We here investigated the association between the methylation status of *AHRR* (cg23576855) in blood leukocyte DNA and the risk of non–Hodgkin lymphoma (NHL), a diverse group of hematologic malignancies that are not associated with tobacco smoking (19). We analyzed prediagnostic blood samples from a previously characterized patient cohort (20) within the population-based Danish Diet, Cancer and Health study. Specifically, we used stratified analyses by time from blood draw to diagnosis to address whether differences in *AHRR* methylation levels could provide a long-term marker of NHL susceptibility or instead reflect a response to tumor growth prior to diagnosis. To compare the overall content of methylated cytosine (5-methylcytosine) in the genome, we measured methylation in long interspersed nucleotide element 1 (LINE-1). Because of the high representation of LINE-1 (∼500,000 copies constituting 17% of the human genome), the mean LINE-1 methylation level is often used as a surrogate measure of global DNA methylation (21).

## Materials and Methods

### Study Population

We applied a case-cohort design within the Diet, Cancer and Health study, which is a Danish prospective cohort study enrolling participants between 1993 and 1997. Almost all men and women ages 50 to 64 years residing in the areas of Copenhagen and Aarhus, who were born in Denmark and free of cancer at time of invitation, were invited to participate, and 57,053 were enrolled (response rate: 35%; ref. 22). All participants completed a self-administered questionnaire concerning health status, family history of cancer, lifestyle, social and reproductive factors as well as a 192 items food frequency questionnaire (FFQ). Physical examinations were performed by trained staff and included anthropometric and blood pressure measurements, and samples of blood, urine, and fat tissue were collected and stored at −180°C. All participants were followed through register linkage via the unique personal identification number from the date of their clinic visit (baseline) to date of cancer diagnosis, death, emigration, or July 31, 2008, whichever came first. Information on vital status and emigration was obtained from the Central Population Registry.

Our study population and procedure for selection of NHL cases and subcohort members were described previously (20). In brief, through linkage with the Danish Cancer Registry (23), we identified 239 cases of NHL during follow-up, and we selected at random a comparison group (subcohort) consisting of 245 cohort members. DNA from blood collected from 480 study participants (239 cases and 241 cohort members) at baseline, that is, before a cancer diagnosis, was available for methylation analysis. The study was approved by the research ethics committee for Copenhagen and Frederiksberg. All participants provided written informed consent at enrollment into the cohort.

### DNA Methylation and SNP Analysis

Approximately 1 μg of genomic DNA was treated with bisulfite using the EZ DNA Methylation Kit (Zymo Research), according to the manufacturer's instructions. DNA recovery after bisulfite conversion was quantified by droplet digital PCR analysis of *MYOD1*, as described previously (24).

The methylation levels of *AHRR* (cg23576855) and LINE-1 were evaluated by pyrosequencing of bisulfite-converted DNA, using the PyroMark PCR Master Mix (Qiagen) with a final MgCl_2_ concentration of 1.5 mmol/L and final primer concentrations of 200 nmol/L. The samples were analyzed using the PyroMark Q24 Instrument (Qiagen), and the results were evaluated using the PyroMark software (Qiagen). Results were included for samples that passed the inbuilt overall quality assessment using standard settings. All experiments included a positive control for methylation [bisulfite-treated, *in vitro* methylated DNA (Universal Methylated DNA Standard, Zymo Research)], a negative control for methylation (bisulfite-treated, whole genome–amplified DNA), and a no-template control.

The methylation status of CpG sites at positions 221 to 305 of LINE-1 was analyzed using PyroMark Q96 CpG LINE-1 (Qiagen), according to the manufacturer's instructions. Results were included if the analysis was successful at both CpG sites, and the mean level across sites was used as the final measure of LINE-1 methylation. Data on a total of 424 samples (208 cases and 216 subcohort members) were available for the final analysis.

Before analysis of *AHRR* (cg23576855) methylation, we excluded carriers (*n* = 48) of the minor allele (A) of the CG>CA SNP at this site (rs6869832), as the CpA dinucleotide cannot be methylated (7). The remaining 376 samples were subjected to pyrosequencing using forward primer AGGATATAGGGGTTGTTTAGGTTA, reverse primer [Btn]-ACAAAACTACCCTCTCAAAAATAAACAAT, and sequencing primer GGGTTGTTTAGGTTATAGATT. Pyrosequencing of *AHRR* was successful in 161 cases and 164 subcohort members.

### Lifestyle and Dietary Factors Recorded at Baseline

Participants were characterized by smoking status (never, former, current smokers). Alcohol consumption and dietary intake were derived from an FFQ. Alcohol was assessed as consumption of beer, wine, and spirits in 12 predefined response categories ranging from “never” to “eight drinks or more per day.” Physical activity was assessed by questions covering the average number of hours per week spent in the past year in leisure time (e.g., jogging, exercising, swimming, and cycling). A diet score was composed of information on energy percentage from fat, red and processed meat, fish, whole grain, and fruits and vegetables. Each item was dichotomized according to the dietary recommendations as suggested previously (25). We grouped the score into adherence to less than two recommendations and two or more.

### Statistical Analysis

The incidence rate ratios (IRR) for NHL were estimated by a Cox proportional hazards (PH) model with age as the underlying time scale. The 95% confidence intervals (CI) were based on the robust estimates of the variance-covariance matrix (26). We used the upper quartile of methylation level as the reference when analyzing the IRRs in association with each of the lower quartiles of methylation level. The *P* value for trend was obtained by introducing the categorical methylation variable based on quartiles as an ordinal linear term. All tests of statistical significance were two sided.

Potential confounding was considered by adjusting the models for age, sex, and educational level. Additional analyses were performed adjusting for smoking status, body mass index (BMI), alcohol consumption, diet score, and physical activity. We analyzed data with increasing level of adjustment, to investigate potential influence of different variables. Model 1: adjustment for age and sex. Model 2: model 1 and additional adjustment for education, BMI, alcohol consumption, diet score, and physical activity. Model 3: model 2 and additional adjustment for smoking status. We *a priori* chose the most extensively adjusted model (model 3) as our main model.

We also analyzed data by time between baseline (blood draw) and diagnosis of NHL using three groups of cases: those with a diagnosis within the first 5 years after baseline, a diagnosis 5 to 10 years after baseline, and a diagnosis more than 10 years after baseline.

We tested the PH assumption of the Cox models for all covariates by a correlation test between the scaled Schoenfeld residuals and the rank order of event time. We detected deviation from the PH assumption for BMI and diet score and therefore included these as strata.

We used the function “cox.zph” to test the PH assumption in the statistical software package R, version 3.3.3. All other analyses were conducted in SAS, version 9.4 (SAS Institute Inc.).

### Data Availability

Data are available upon request to the Danish Cancer Society (dchdata@cancer.dk).

## Results

LINE-1 methylation levels (a validated surrogate marker for global DNA methylation) were measured in leukocyte DNA from 208 study participants who developed NHL at a median of 8.5 years (range, 0.2–13.4 years) after blood draw, as well as a subcohort of 216 participants. *AHRR* (cg23576855) methylation levels could be assessed in 161 and 164 of these participants, respectively. Baseline characteristics of cases and subcohort members are shown in Table 1. Cases were older than subcohort members but cases and subcohort members were similar with respect to all other variables shown in Table 1, including sex, educational level, smoking status, BMI, alcohol consumption, physical activity, and diet score. The characteristics of the participants eligible for LINE-1 and *AHRR* methylation analysis, respectively, were similar except for a higher proportion of obese among those eligible for *AHRR* analysis (Table 1).

**TABLE 1:** Baseline sociodemographics and lifestyle characteristics of the study population

	Participants with information on LINE-1 methylation, *n* (%)		Participants with information on *AHRR* methylation, *n* (%)	
Variable	Cases	Subcohort	Cases	Subcohort
Overall	208	216	161	164
Age				
≤60 years	143 (69)	166 (77)	111 (69)	124 (76)
>60 years	65 (31)	50 (23)	50 (31)	40 (24)
Sex				
Women	98 (47)	103 (48)	80 (50)	77 (47)
Men	110 (53)	113 (52)	81 (50)	87 (53)
Educational level				
<8 years	73 (35)	63 (29)	58 (36)	48 (29)
8+ years	135 (65)	153 (71)	103 (64)	116 (71)
Smoking status				
Never-smokers	70 (34)	82 (38)	51 (32)	59 (36)
Previous smokers	62 (30)	61 (28)	52 (32)	47 (29)
Smokers	76 (37)	73 (34)	58 (36)	58 (35)
BMI				
≤25.0 kg/m^2^	85 (41)	93 (43)	66 (41)	73 (45)
25.1–30.0 kg/m^2^	84 (40)	94 (44)	59 (37)	74 (45)
>30.0 kg/m^2^	39 (19)	29 (13)	36 (22)	17 (10)
Alcohol				
≤12/24 drinks per week	126 (61)	128 (59)	97 (60)	102 (62)
>12/24 drinks per week	82 (39)	88 (41)	64 (40)	62 (38)
Physical activity				
<30 minutes per day	124 (60)	136 (63)	98 (61)	105 (64)
30+ minutes per day	84 (40)	80 (37)	63 (39)	59 (36)
Diet score				
0–1 points	147 (71)	133 (62)	110 (68)	100 (61)
2+ points	61 (29)	83 (38)	51 (32)	64 (39)

Table 2 shows *AHRR* and LINE-1 methylation levels by case/subcohort status and sociodemographic and lifestyle factors. *AHRR* methylation levels were lower in men, smokers, persons with lower physical activity level, and persons with lower diet score (less healthy diet) but similar with respect to age, educational level, BMI, and alcohol intake. There were no associations between LINE-1 methylation levels and any of the baseline characteristics shown in Table 2.

**TABLE 2:** Median methylation levels in NHL cases and subcohort, by baseline sociodemographics and lifestyle characteristics

	LINE-1		*AHRR*	
Variable	Cases (*n* = 208)	Subcohort (*n* = 216)	Cases (*n* = 161)	Subcohort (*n* = 164)
Overall	68.1	68.3	77.1	79.6
Age				
≤60 years	68.1	68.3	77.6	78.4
>60 years	68.2	68.4	76.9	81.0
Sex				
Women	68.2	68.2	83.1	81.3
Men	68.1	68.4	72.5	76.3
Educational level				
<8 years	68.2	68.0	75.3	78.4
8+ years	68.1	68.5	78.0	79.7
Smoking				
Never-smokers	68.1	68.3	84.5	83.9
Previous smokers	67.5	68.5	80.6	81.0
Smokers	68.7	68.2	51.4	60.0
BMI				
≤25.0 kg/m^2^	68.2	68.6	75.0	76.8
25.1–30.0 kg/m^2^	68.1	68.0	81.8	80.4
>30.0 kg/m^2^	68.1	68.6	76.9	80.9
Alcohol				
≤12/24 drinks per week	68.1	68.3	76.7	80.6
>12/24 drinks per week	68.3	68.4	79.2	76.8
Physical activity				
<30 minutes per day	68.2	68.4	72.5	76.8
30+ minutes per day	68.1	68.2	83.1	81.0
Diet score				
0–1 points	68.1	68.4	75.0	77.0
2+ points	68.1	68.1	83.1	81.6

We found no statistically significant association between *AHRR* or LINE-1 methylation levels, and NHL. The IRR for the lowest versus highest quartiles of *AHRR* methylation was 2.17 (95% CI, 0.90–5.21) in the fully adjusted model (Table 3). The IRR for the lowest versus highest quartiles of LINE-1 methylation was 1.34 (95% CI, 0.77–2.34) in the fully adjusted model (Table 4).

**TABLE 3:** IRRs for NHL and 95% CI in association with *AHRR* methylation

		IRR (95% CI)		
	*n* (cases/subcohort)	Model 1^b^	Model 2^c^	Model 3^d^ (main model)
Methylation levels^a^				
>84.6%	40/41	1.00	1.00	1.00
84.5%–78.3%	37/44	0.84 (0.45–1.56)	0.84 (0.45–1.57)	0.86 (0.46–1.61)
78.2%–62.2%	37/44	0.85 (0.45–1.59)	0.83 (0.44–1.58)	0.84 (0.43–1.66)
<61.8%	47/35	1.54 (0.81–2.92)	1.80 (0.92–3.53)	2.17 (0.90–5.21)
*P*_trend_		0.22	0.14	0.30

^a^Cut-off points corresponding to quartiles of the distribution among the total sample.

^b^Adjusted for age and sex.

^c^Model 1 + educational level, BMI (strata), alcohol consumption, diet score (strata), and physical activity.

^d^Model 2 + smoking.

**TABLE 4:** IRRs for NHL and 95% CI in association with LINE-1 methylation

		IRR (95% CI)		
	*n* (cases/subcohort)	Model 1^b^	Model 2^c^	Model 3^d^ (main model)
Methylation levels^a^				
>69.5%	47/58	1.00	1.00	1.00
68.3%–69.5%	52/54	1.19 (0.68–2.08)	1.28 (0.73–2.24)	1.31 (0.75–2.30)
66.8%–68.2%	54/52	1.30 (0.75–2.25)	1.31 (0.75–2.31)	1.34 (0.76–2.35)
<66.8%	55/52	1.26 (0.72–2.18)	1.33 (0.76–2.32)	1.34 (0.77–2.34)
*P*_trend_		0.40	0.32	0.32

^a^Cut-off points corresponding to quartiles of the distribution among the total sample.

^b^Adjusted for age and sex.

^c^Model 1 + educational level, BMI (strata), alcohol consumption, diet score (strata), and physical activity.

^d^Model 2 + smoking.

Table 5 shows associations between *AHRR* methylation levels and NHL according to time to diagnosis (i.e., the time interval between blood draw and diagnosis of NHL). Overall, the IRR for *AHRR* hypomethylation (lowest vs. other quartiles) was 2.52 (95% CI, 1.24–5.15). In the fully adjusted model, the IRR was 4.50 (95% CI, 1.62–12.50) for cases diagnosed <5 years after blood draw and 7.04 (95% CI, 2.36–21.02) for cases diagnosed 5–10 years after blood draw. There was no association for cases diagnosed >10 years after blood draw (IRR, 0.56; 95% CI, 0.21–1.45). Also, there was no association between LINE-1 methylation levels and NHL when data were stratified according to time to diagnosis (Table 6).

**TABLE 5:** IRRs for NHL and 95% CI in association with *AHRR* methylation levels, stratified by years from baseline (blood draw) to diagnosis

		IRR (95% CI)			
		Full follow-up 1^b^	0–5 years after baseline^b^	5–10 years after baseline^b^	10+ years after baseline^b^
Methylation levels^a^	*n* (cases/subcohort)	161/164	47/164	66/160	48/147
≥61.8%	114/129	1.00	1.00	1.00	1.00
<61.8%	47/35	2.52 (1.24–5.15)	4.50 (1.62–12.50)	7.04 (2.36–21.02)	0.56 (0.21–1.45)

^a^Cut-off point corresponding to the 25th percentile of the total sample distribution.

^b^Adjusted for age, sex, educational level, smoking, BMI (strata), diet score (strata), physical activity, and alcohol consumption.

**TABLE 6:** IRRs for NHL and 95% CI in association with LINE-1 methylation levels, stratified by years from baseline (blood draw) to diagnosis

		IRR (95% CI)			
		Full follow-up 1^b^	0–5 years after baseline^b^	5–10 years after baseline^b^	10+ years after baseline^b^
Methylation levels^a^	*n* (cases/subcohort)	208/216	57/216	92/210	59/193
≥66.8%	153/164	1.00	1.00	1.00	1.00
<66.8%	55/52	1.10 (0.71–1.72)	0.60 (0.28–1.32)	1.29 (0.75–2.23)	1.38 (0.75–2.52)

^a^Cut-off point corresponding to the 25th percentile of the total sample distribution.

^b^Adjusted for age, sex, educational level, smoking, BMI (strata), diet score (strata), physical activity, and alcohol consumption.

## Discussion

In this study, we found that lower levels of *AHRR* (cg23576855) methylation in peripheral blood leukocytes were associated with a future diagnosis of NHL in a general Danish population. The greatest divergence from subcohort *AHRR* methylation levels was observed among cases with shorter time between blood draw and diagnosis, whereas there was no association among cases diagnosed more than 10 years after blood draw. We found no statistically significant associations between LINE-1 methylation levels and NHL, neither overall nor when stratified by years from blood draw to diagnosis.

Previous studies have shown that low *AHRR* (cg05575921) methylation is a strong marker of tobacco smoke exposure (7–13). We confirmed this association for *AHRR* (cg23576855) in both cases and subcohort members, and found *AHRR* hypomethylation to also be associated with male sex and lifestyle characteristics such as lower physical activity level and a less healthy diet. Adjustment for all these factors did not affect the association between *AHRR* hypomethylation and NHL, indicating that residual confounding is unlikely to explain this observation. Moreover, the absence of a correlation between LINE-1 methylation and NHL indicates that the reduced levels of *AHRR* methylation observed in individuals diagnosed with NHL later in life are not attributed to overall DNA demethylation.

The results from our study in NHL are consistent with reverse causality in which the observed differences in *AHRR* methylation reflect a response to tumor growth preceding clinical diagnosis. First, tobacco smoking is not a known risk factor for NHL (27), and thus smoking-induced methylation changes could not explain the observed effect. Second, if lower levels of *AHRR* methylation were a long-term marker of NHL susceptibility, methylation levels would be expected to be independent of time to diagnosis. In contrast, we observed a strong association of *AHRR* methylation with time to diagnosis. A recent study found an association between a methylation signature comprising 135 CpG sites and risk of NHL (28). However, this study did not explore the time element, nor did it address the matter of causality.

Strengths of this study are the availability of blood samples prospectively collected from apparently healthy persons in a general population setting, as well as the long cancer follow-up after blood draw. Furthermore, the Danish registries are virtually complete, providing reliable information on cancer incidences. We also had detailed information on the participants’ characteristics at baseline, allowing us to adjust for multiple potential predictors of DNA methylation levels or NHL. A limitation of our study is the use of only one sample per subject; investigations involving longitudinal blood samples would provide more robust data for capturing the temporal dynamics of DNA methylation.

In conclusion, our results provide evidence of a link between *AHRR* hypomethylation in blood leukocytes and future development of NHL. Although additional studies are warranted to confirm these results, our study suggests that *AHRR* hypomethylation may precede NHL as a response to tumor development. This could inspire similar studies in a broader context and should be taken into account before clinical implementation of *AHRR* methylation as a biomarker, for example to improve eligibility assessment for lung cancer screening (15).
